# Influence of Surface-Modified Montmorillonite Clays on the Properties of Elastomeric Thin Layer Nanocomposites

**DOI:** 10.3390/ma16041703

**Published:** 2023-02-17

**Authors:** Adam Olszewski, Aleksandra Ławniczak, Paulina Kosmela, Marcin Strąkowski, Aleksandra Mielewczyk-Gryń, Aleksander Hejna, Łukasz Piszczyk

**Affiliations:** 1 Department of Polymer Technology, Chemical Faculty, Gdańsk University of Technology, G. Narutowicza St. 11/12, 80-233 Gdańsk, Poland; 2 Department of Metrology and Optoelectronics, Faculty of Electronics, Telecommunications and Informatics, Gdańsk University of Technology, G. Narutowicza St. 11/12, 80-233 Gdańsk, Poland; 3 Faculty of Applied Physics and Mathematics, Gdańsk University of Technology, G. Narutowicza St. 11/12, 80-233 Gdańsk, Poland; 4 Institute of Materials Technology, Poznan University of Technology, Piotrowo 3, 61-138 Poznań, Poland

**Keywords:** nanocomposite, montmorillonite, polyurethane, thin layers, surface modification

## Abstract

In recent years, polyurethane nanocomposites have attracted more attention due to the massive demand for materials with increasingly exceptional mechanical, optical, electrical, and thermal properties. As nanofillers have a high surface area, the interaction between the nanofiller and the polymer matrix is an essential issue for these materials. The main aim of this study is to validate the impact of the montmorillonite nanofiller (MMT) surface structure on the properties of polyurethane thin-film nanocomposites. Despite the interest in polyurethane–montmorillonite clay nanocomposites, only a few studies have explored the impact of montmorillonite surface modification on polyurethane’s material properties. For this reason, four types of polyurethane nanocomposites with up to 3% content of MMT were manufactured using the prepolymer method. The impact of montmorillonites on nanocomposites properties was tested by thermogravimetric analysis (TGA), dynamic mechanical analysis (DMA), contact angle measurement, X-ray diffraction (XRD), and optical coherence tomography (OCT). The results showed that chemical and physical interactions between the polymer matrix and functional groups on the montmorillonite surface have a considerable impact on the final properties of the materials. It was noticed that the addition of MMT changed the thermal decomposition process, increased *T*_2%_ by at least 14 °C, changed the hydrophilicity of the materials, and increased the glass transition temperature. These findings have underlined the importance of montmorillonite surface structure and interactions between nanocomposite phases for the final properties of nanocomposites.

## 1. Introduction

The growing impact of polymer composite materials has been observed in the worldwide material markets. Continuously increasing interest is caused by the capability to manufacture materials with significantly improved properties due to the synergistic effect between composite phases [1,2,3,4]. The matrix of polymer composites may be composed of a thermosetting or thermoplastic polymer, which directly determines the properties, processing method, and future material application. The dispersed phase consists of inorganic compounds like silicates, phyllosilicates, particles, and fiber systems (fabrics, mats, short and medium-length staple fibers, and long fibers) or organic compounds like cellulose fibers, wood flour, three-dimensional hybrid nanofillers, and allotropic species of coal, e.g., carbon nanotubes [5]. An interesting type of material, due to the size of the dispersed phase, are polymer nanocomposites (PNCs). These materials contain nanofiller, which is quantified on a nanometric scale [6]. For a better understanding of the used nomenclature, the most important abbreviations are presented in Table 1.

In recent years, an increasing interest in polymer nanocomposites has been noticed globally, which might be proven by numerous publications [1,2,3,4,7,8,9,10,11]. Nanocomposites enable materials to be synthesized with improved mechanical, optical, electrical, thermal, and barrier properties in comparison with their macro equivalents [12,13]. Moreover, the amount of filler required to achieve the same effect of reinforcement is smaller (usually up to 3 wt%). All of the above-mentioned effects may be associated with an increase in matrix–nanofiller interaction surface, chemical reactivity, adhesion forces, and catalytic activity with simultaneous downsizing of filler [14]. The indicated characteristics of nanocomposites result not only from the size and filler structure but also strongly depend on the level of dispersion and shape or volume of the sample. Due to high surface area, nanofillers lead to the obtaining of specific properties at the nanoscale—such as surface plasmon resonance or quantum dot phenomena [15]. Depending on the filler distribution, systems like the isotropic, anisotropic, and orthotropic varieties are distinguished.

Among various types of nanocomposite matrixes, polyurethane (PU) matrices are widely used in research [8,9,16,17,18,19]. Polyurethanes are synthesized through a reaction between a compound with isocyanate (-NCO) groups and substances containing at least two hydroxyl groups (-OH)—polyols. As a result of this reaction, urethane bonds are obtained. Polyurethanes are materials with distinctive segmentary structures with soft and hard segments. The composition of the segments has a direct influence on the physical properties of the PU system. Generally, it is observed that with an increased amount of hard segments, the toughness, absorption resistance, and Young’s modulus increase. The hard segments of polyurethane are mainly composed of isocyanates and low-molecular-weight chain extenders. Furthermore, with an increasing number of soft segments, parameters such as elongation at break, flexibility, and low-temperature resistance increase. The structure of PU soft segments depends on the structure of the polyols—their functionality, branching, and molecular mass. Furthermore, as the content of the hard segments increases, the material will change from flexible and rubber-like to rigid and tough plastic [20]. Jing et al. [21] concluded that urea units in hard segments actively influence the mechanical properties (especially tear and tensile strength) at higher temperatures of polyurethane elastomers based on diethyl oxalate and amino-terminated polypropylene oxide. Meanwhile, amide units in soft segments weaken intermolecular interactions between soft and hard segments, resulting in microphase separation and increased tear strength at elevated temperatures. The appropriate synthesis and balancing of the hard segment content also allow for changing the glass transition temperature of the PU [22]. This modification allows for the application of these materials in various conditions, for example, in medicine (bone regenerative medicine [23]), the coating industry (corrosion-resistant coating [24]), agriculture (controlled-release fertilizer [25]), and the automotive industry (nonwoven nice-in-touch automotive interior coatings [26]).

During the material design process to achieve the preferred properties of the composite with a polyurethane matrix, it is necessary to add adequate filler. The literature shows a popular trend of manufacturing PU nanocomposites with montmorillonite clays (MMT) [2,8,9,11,16,17,18,19]. MMT belongs to the family of 3D nanolayered silicates with a 2:1 package type of layers (two tetrahedral layers and one octahedral layer) [27]. Due to the very high surface–volume ratio of the nanoclay plates (with a thickness of one nanometer and several hundred nanometers in dimension) of the MMT clay, their surface properties contribute to the bulk properties of the nanocomposites. The layered structure consists of silicate SiO_4_ tetrahedrons with oxygen atoms in the corners and a silicon atom in the center of the tetrahedron crystal [28].

In terms of chemistry, montmorillonite is defined as M_x_(Al_4−x_Mg_x_)Si_8_O_20_(OH)_4_, where M is a cation that has an oxidation state of +1 (e.g., Na^+^, H^+^) and X amounts to 0.5–1.3 [29]. Isomorphous substitution of Si^4+^ and Al^4+^ by Mg^2+^ and Fe^2+^, and less frequently Mg^2+^ by Li^+^, is possible, resulting in a negative charge of the layered package. The unpaired electron is balanced by adding an alkaline monovalent cation such as Na^+^, K^+^, Li^+^, H^+^, or an alkaline earth cation, usually Ca^2+^. The result is an intralayering of cations that neutralize the electrical charge. Depending on the cations used, sodium (Na-MMT), calcium (Ca-MMT), and hydrogen (H-MMT) montmorillonites can be distinguished. Naturally, montmorillonite is a hydrophilic substance. Thus, unwanted limitations of polymer intercalation due to emerging aggregations may occur while obtaining a polymer nanocomposite. To achieve an optimal dispersion of silicate in polymers, it is necessary to modify montmorillonite with various cationic molecules to decrease the surface energy of the clay, which enhances the distance between the layers and improves the nanofiller dispersion in the matrix [28,30]. In recent years, researchers replaced intralayer cations with amino compounds, thiols, and silanes, which improved clay dispersion in polymers, maximized heavy metal cations’ adsorption capacities, increased thermal stability, and improved the tensile strength and modulus [31,32,33,34,35,36,37]. Modification by organic ions also impacts clay hydrophobicity, where the exact influence depends on cationic characteristic [38,39]. According to the size and length of the alkyl chains of the surfactant, the interlayer space of MMT can be more filled, resulting in less possibility of absorption water molecules. 

The influence of modified MMT on polyurethane/montmorillonite nanocomposites was studied by Xu et al. [16], who obtained highly exfoliated montmorillonite clay-reinforced thermoplastic polyurethane elastomers by in situ solution polymerization. Adding 1 wt% of MMT modified by 4,4-methylene diphenyl diisocyanate (MDI) resulted in increases in the Young’s modulus of 46%, tensile strength by 36%, and elongation at break by 40% in comparison to the empty polyurethane matrix. The increase in MDI-MMT T_10%_ (decomposition temperature of 10% by weight of the material) and T_50%_ (decomposition temperature of 50% by weight of the material) was due to the blocking effect of MMT. 

Similar effects were observed by Qiao et al. [9], who modified montmorillonite with cetryltrimethylammonium bromide (CMMT) and obtained PU/CMMT nanocomposites by in situ polymerization. The authors studied the mechanical properties and noticed that the tensile and tear strength of the PU/CMMT composites increased by almost 8% and 10%, respectively, with the increase in the CMMT content from 2 wt% to 3 wt%. In the case of samples with higher CMMT loading, the tensile and tear strength increased only by 3.87% and 2.49%, respectively. This effect is related to the limited dispersion of the silicate layers in the PU matrix.

Zhao et al. [28] also studied the addition of montmorillonite to a polyurethane elastomer. Modified MMT was obtained by intercalating chlorhexidine acetate (CA) into interlayers of MMT (CA-MMT). The functionalized MMT caused improvement in the thermal stability, reaching 285.8 °C in comparison to the pure PU (232.3 °C). Modification by chlorhexidine acetate (an antimicrobial agent) allowed the obtaining of PU/CA-MMT, which is characterized by good resistance to bacterial adhesion and antibacterial ability. The CA-MMT filler increased the activation energy of thermal oxidation, resulting in improved resistance to aging.

Analyzing the available scientific literature about polyurethane/montmorillonite nanocomposites, a huge interest in these materials can be noticed. On the other hand, a significant gap in knowledge related to the influence of montmorillonite modification on thin polyurethane elastomer nanocomposites can be noticed. Moreover, only a few studies explored impact of montmorillonite surface structure on properties of nanocomposites. This effect is especially important for materials which have to meet strict requirements for possible application. The present study aims to fill these knowledge gaps. In this paper, we examined the impact of the type and amount of four different functionalized montmorillonites on properties of elastomeric thin-layer polyurethane nanocomposites. The properties of manufactured PU/MMT films with 0.5–3.0 wt% filler content were analyzed by thermogravimetric analysis (TGA), dynamic mechanical analysis (DMA), contact angle measurement, X-ray diffraction analysis (XRD), and optical coherence tomography (OCT). 

## 2. Materials and Methods

### 2.1. Materials

The information on the substrates applied in this study is presented in Table 2.

### 2.2. Manufacturing of PU/MMT Nanocomposite Thin Films

Polyurethane materials and nanocomposites were manufactured using the two-step method (prepolymer method). During the first step, polyol Polios 55/20 (α,ω-oligo(ethylene-butylene adipate) with *M_n_*~2000 g/mol was dried for 2 h under reduced pressure (0.1 Bar–10 kPa). After that, the calculated amount of methylene 4,4-diphenyl diisocyanate was added to the reactor to obtain a urethane prepolymer with 8% free NCO content. The reaction was conducted at a temperature of ~80 °C until the stabilization of the NCO free group content (2 h). The titration of free NCO content was conducted according to ASTM D 2572-97 [40]. The final free NCO content of the synthesized prepolymer was 8.04%. The second synthesis step started with the dispersion of dry montmorillonite clays (24 h at 100 °C) in prepolymer using a homogenizer (3000 rpm for 5 min) in an ultrasound bath. Next, a chain extender (BDO) with a catalyst (1,4-diazabicyclo [2.2.2]octane) was added to the prepolymer and mixed again (isocyanate index = 1.05). The obtained mixture was degassed under reduced pressure (0.1 Bar–10 kPa), poured onto Teflon plates, and aligned with a stainless-steel applicator (200 μm). Manufactured samples were conditioned at 100 °C for 24 h. The neat PU thin film and 16 sets of PU thin film nanocomposites with the addition of 0.5–3.0 wt% nanofiller were manufactured for the tests. The samples are coded **X% YY**, where **X%** is the amount of nanofiller and **YY** is the abbreviation for the nanofiller from Table 2.

### 2.3. Characterization

Water and diiodomethane contact angles were measured using a Rame-Hart 90-U3-PRO goniometer from Rame-Hart Instrument Co. (Succasunna, NJ, USA). Thin film samples (20 mm × 20 mm × ≈ 0.2 mm) were cut off and located on the goniometer. Using the Drop Image Pro (v. 3.19.1.0) software, a drop of liquid was placed on a flat part of the sample, and contact angles were measured right after application on the sample surface and after two minutes (only for water droplets). The measurement was repeated three times for each sample. The measurement was conducted at room temperature (21 °C).

The thermal stability of the manufactured polyurethane (PU) thin layers was determined by thermogravimetric analysis (TGA). The measurements were carried out using a Netzsch TG 209 F3 apparatus (Selb, Germany). The measurements of 10 ± 1 mg samples were made in the temperature range of 30 to 800 °C and at a heating rate of 10 °C/min in a nitrogen-constant flow.

The dynamic mechanical analysis (DMA) of the polyurethane thin films was conducted using a DMA Q800 TA Instrument (New Castle, DE, USA). Samples were analyzed in tension film mode with a frequency of 1 Hz. Measurements were performed in the temperature range of −100 to 100 °C with a heating rate of 3 °C/min. Beam-shaped samples with dimensions of 20 mm × 10 mm × ≈ 0.2 mm were used.

X-ray diffraction (XRD) patterns were recorded using an X’Pert PRO (MPD) PANalytical X-ray diffractometer (Malvern Panalytical, Malvern, UK) with a copper anode lamp (CuK_α1_, λ = 0.1546 nm, 40 kV, 30 mA) at a scanning rate of 0.25°/s^−1^ and in a 2θ in range from 10° to 90°.

**Figure 1:**
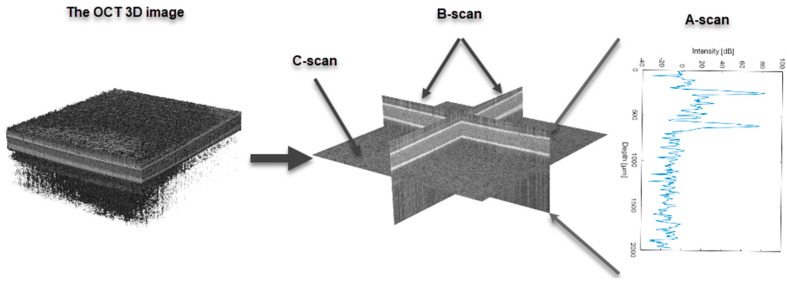
The optical coherence tomography imaging planes.

In this study, optical coherence tomography (OCT) delivered volumetric information about the tested samples (DUT—the device under test) based on the backscattered light intensity from their inner structures. To introduce the imaging planes of the volumetric data, one may distinguish a B-scan—a standard two-dimensional (2D) cross-sectional tomographic image; C-scan—the 2D image of the surface in the perpendicular plane to the B-scan at the specified depth inside the DUT; and A-scan—a single depth-resolved line in the B-scan (Figure 1) [41]. An IVS-2000-PS-OCT system (Santec Inc, Fukuoka, Japan) was used for the experiments. Its features are summarized in Table 3.

Based on the OCT data, each sample was evaluated to estimate its thickness, optical absorption, and scattering features as an extinction coefficient normalized to the sample refractive index (*µ*_e_). The thickness was measured by calculating the distance between the highest peaks in A-scans, which correspond to the light scattering and reflection from the top and bottom sample surface. The obtained values represented the optical thickness, where *d* is the actual geometric thickness, and *n* is the refractive index. The extinction coefficient was estimated based on the Bouguer–Lambert–Beer law, which expresses the relation of the optical beam transmitted through the scattering and absorption device to the intensity of the illuminating beam. The expression of the Bouguer–Lambert–Beer law according to the optical thickness of the DUT is given as Equation (1):

(1) $$I=I_{0}e^{-{µ}_{e}\cdot d_{n}}$$

where *I* is the intensity of the output beam, *I*_0_ is the intensity of the illuminating beam, *d_n_* is the optical thickness of the tested device, and *µ*_e_ is the extinction coefficient normalized to the refractive index of the sample. The *µ*_e_ was estimated from the absorbance calculated from the A-scans. Due to the A-scans’ logarithmic (dB unit) scale, the absorbance is given as a difference between peak values representing the optical reflection from the top and bottom surfaces. From this, the *µ_e_* can be estimated directly. However, it is noticed that the obtained value is doubled due to the double passage of the light through the tested sample. The calculations were made according to Equation (2):

(2) $${\text{µ}}_{e}=\frac{(I_{1}-I_{2})/20)*\ln\left(10\right)}{2*d_{n}}$$

where *I*_1_ (dB) and *I*_2_ (dB) are the recorded intensity of the backscattered light from the top and bottom sample surfaces (in dB units), respectively, and *d_n_* is the thickness measured by the OCT method.

## 3. Results

### 3.1. Thermogravimetric Analysis (TGA)

**Figure 2:**
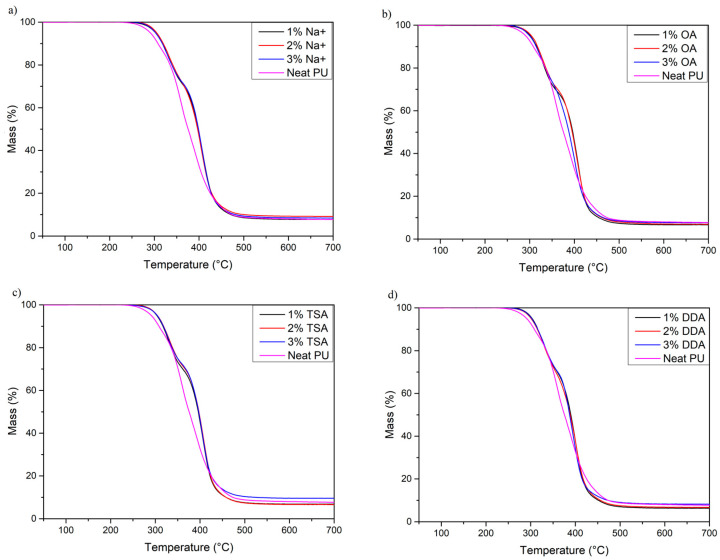
Thermogravimetric (TG) curves of neat PU and manufactured nanocomposites. (**a**) Na+ samples; (**b**) OA samples; (**c**) TSA samples; (**d**) DDA samples.

**Figure 3:**
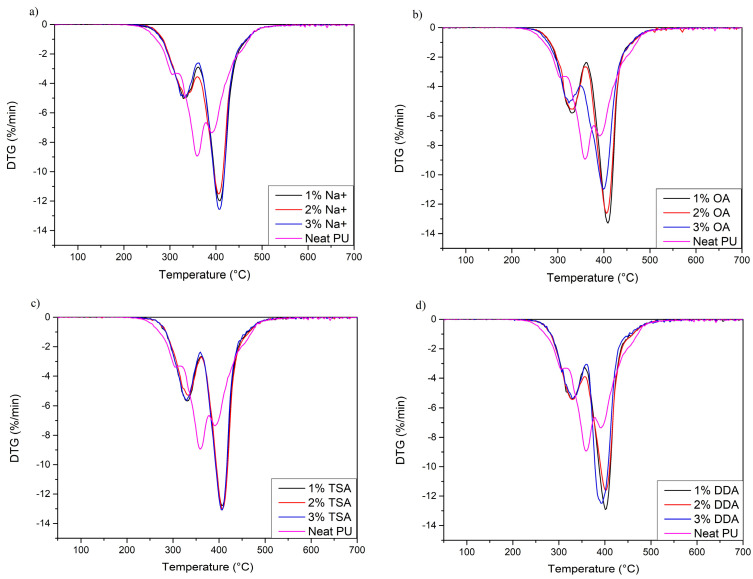
DTG curves of neat PU and manufactured nanocomposites. (**a**) Na+ samples; (**b**) OA samples; (**c**) TSA samples; (**d**) DDA samples.

As the addition of nanofiller strongly affects the thermal stability of PU materials, thermogravimetric analysis was conducted. The results are presented in Figure 2 and Figure 3, and Table 4. When analyzing the results, it can be concluded that the *T*_2%_ of the nanocomposites increased significantly compared to neat PU. The *T*_2%,_ commonly claimed as the thermal stability of PU materials, increased from around 271 °C for neat PU to around 285–290 °C for all PU/MMT nanocomposites (a change of 25–30 °C). An equally important change was observed for the *T*_5%_ and *T*_50%,_ which increased by 10–15 °C and 20–25 °C, respectively. The observed increase in thermal stability in a wide range may be related to strong interactions between functional groups of polyurethane chains and nanofiller or chemical bonding between two phases of composites, as shown by Xiong et al. [44]. In our case, an example of strong interaction and chemical bonding which increase thermal stability may occur between OA and DDA montmorillonites. Generally, amines may react with isocyanates, which leads to the generation of urea groups [45]. In addition, the thermal stability may also be increased as a result of the limitation of heat and mass transfer. For this reason, the decomposition process is slower than for neat polyurethane [46]. Moreover, the increase in thermal stability may also be enhanced by the proper distribution and exfoliation of nanofiller in the PU matrix, which increases the surface of interaction between both phases. These results are in line with those obtained by Tien et al. [47].

Moreover, it can be noticed that neat PU decomposes in a three-step process. The first peak (*T*_max1_) around 304 °C can be attributed to the decomposition of rigid segment parts, consisting of allophanates and biuret groups [48]. The presence of these groups in the structure may be related to the excess of isocyanate groups during the synthesis of PU nanocomposites (NCO/OH ratio = 1.05). The second degradation step with a maximum of 359.9 °C may be associated with the dissociation of urethane bonds and partial degradation of soft segments that have not undergone phase separation. The degradation of soft segments at this temperature was confirmed by thermogravimetric analysis of Polios 55/20, the temperature of maximal degradation of which is around *T*_max_ = 355 °C (curve available in Supplementary Materials). As a result of this process, several different compounds, such as polyols, isocyanates, carbon dioxide, and amine derivatives, may be formed. The third degradation step may result from the degradation of soft segments that have undergone phase separation. These segments are made up of long chains of polyester polyol (Polios 55/20). It can be noticed that this peak is significantly lower than for nanocomposites, which is evidence of lower phase separation.

However, PU nanocomposites degrade in a two-step process regardless of the amount of MMT. Two-step decomposition usually occurs with segmented polyurethane materials and confirms good phase separation [49]. The first peak with a maximum degradation rate of ~330 °C may be due to the degradation of hard segments of PU, leading to the formation of amines, low-molecular-weight components, and carbon dioxide [50]. The shift of *T*_max2_ to lower temperatures may be caused by the higher degree of interfacial separation between soft and hard segments, which may be caused by interactions of both phases with montmorillonite plates [51]. Finally, soft segment degradation occurs with *T*_max3_ at ~400 °C. The shift in soft segment degradation temperature to higher temperatures may be due to stabilization of the soft segment structure by physical and chemical interactions between soft segments and montmorillonites. Moreover, this effect may be enhanced by increased microphase separation in the PU structure. Similar results were obtained in the experiment by Datta et al. [52].

**Figure 4:**
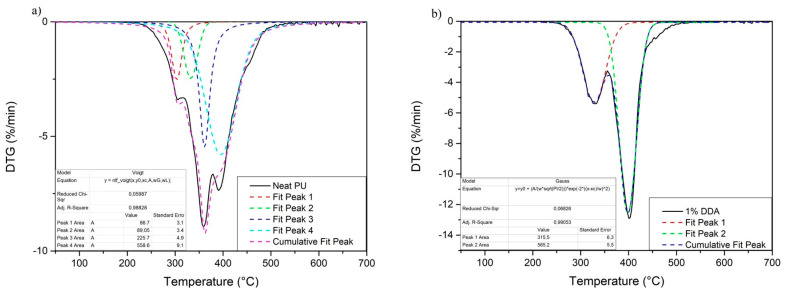
Deconvolution of DTG curves of neat PU and 1% DDA nanocomposite. (**a**) Neat PU; (**b**) 1% DDA sample.

To determine the percentage intensity of each stage of degradation, DTG curves were deconvoluted using the Origin 2021 software. Examples of deconvolution results are shown in Figure 4, and results of calculations are presented in Table 4. The area under the curves was calculated, and the percentage content of rigid segments was estimated similarly to the results obtained by Moo-Espinosa [50]. In the case of DTG curves of nanocomposite samples presented in Figure 4a, the Gaussian calculation method was used. Due to the high degree of phase separation, the values are slightly lower than theoretical amount (≈38.1% of hard segments) which results from the composition of the material. The obtained value may be lower due to incomplete separation of the rigid segments. For the sample without filler presented in Figure 4b, due to the low degree of phase separation, the Gaussian method failed. Instead of this method, the Voigt calculation method was used. It should be noted that, because of the high inaccuracy of the determination of this parameter (despite the high *R* obtained), the result should be regarded as an approximation, as complete peak separation is not possible and a degradation process of a few structures may occur at the same time. It was assumed that the peak from the degradation of well-separated rigid segments occurs around 330 °C. Moreover, this artificially determined peak should not be treated as an additional stage of degradation.

### 3.2. Dynamic Mechanical Analysis (DMA)

**Figure 5:**
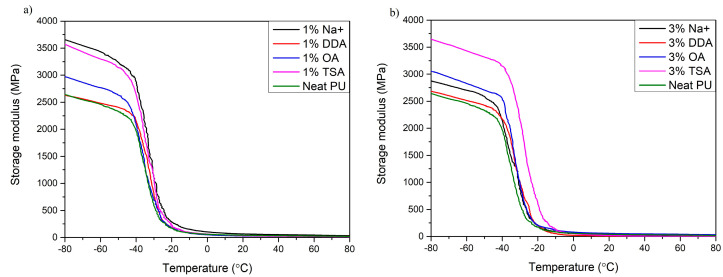
The storage modulus of synthesized nanocomposite thin films. (**a**) Samples with 1% addition of MMT; (**b**) Samples with 3% addition of MMT.

**Figure 6:**
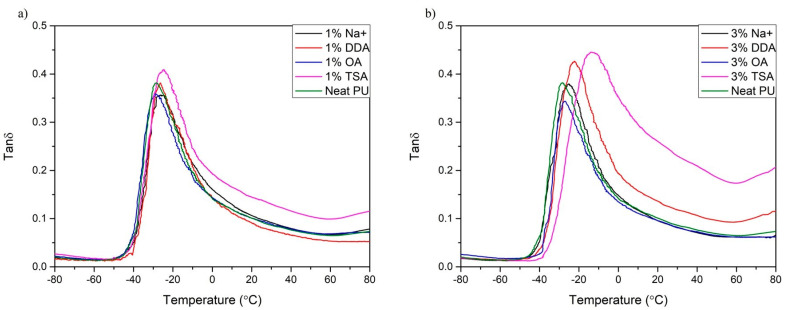
The damping factor of synthesized nanocomposites thin films. (**a**) Samples with 1% addition of MMT; (**b**) Samples with 3% addition of MMT.

A dynamic mechanical analysis, or DMA, was carried out to determine the effect of MMT on thermomechanical properties and the strengthening effect. The storage modulus (*E*’), the loss modulus (*E*”), and the damping factor (tan *δ*) were determined. Based on these parameters, the occurrence of molecular mobility transitions, such as the glass transition temperature (*T*_g_), can be determined. The results of the DMA are presented in Figure 5 and Figure 6, and Table 5.

All materials analyzed show a typical decrease in loss modulus, which is attributed to the glass transition of the polymer matrix [53]. Analyzing the storage modulus at different temperatures (*E*’ at *T*_1_ and *T*_2_), it can be noticed that in the glassy state (below glass transition temperature (*T*_g_), all nanocomposites show a higher storage modulus, which indicates the strengthening effect of the nanofiller [46]. These results align with those obtained by Xu et al. [35], where the addition of MMT nanofiller significantly influences PU’s elastic properties by restricting the movement of PU chains. Moreover, after exceeding the glass transition temperature, the change in the dynamic mechanical performance of all samples is insignificant due to the unblocking of PU chains’ mobility [46].

Moreover, DMA provides not only information on the stiffness of materials but also information on the glass transition temperature (*T*_g_). It can be determined from the onset of the *E’,* the maximum of the peak *E*”, or the maximum peak of tan *δ*. In this work, *T*_g_ was determined from the max peak of tan *δ*. All samples with the addition of MMT have a higher glass transition temperature compared to the polyurethane matrix. A similar relationship was observed by Xu et al. [16]. Nanocomposites require higher temperatures to exceed the Gibbs free energy and achieve a rubbery state. This may indicate a higher structural cross-linking density of the nanocomposites. The glass transition temperature is between −28.37 °C and −14.43 °C. The amount of MMT in the composites also affected the *T*_g_ value. With higher amounts of MMT, the *T*_g_ values shift toward higher temperatures. The most significant effect is observed in Figure 6b for the samples containing DDA and TSA MMT. This effect may be due to interactions and chemical reactions between PU components and the montmorillonite clay and the potential catalytic effect of TSA MMT and DDA MMT on the polymerization reaction [54].

### 3.3. Contact Angle Measurement

The impact of MMT on the hydrophilic and hydrophobic nature of nanocomposites was investigated by measuring the water contact angle at t = 0 min (WCA_0_) and t = 2 min (WCA_2_), as well as the diiodomethane contact angle (ICA). As a result, the surface energy, adhesion work, and dispersive and polar components of the surface energy were calculated and are presented in Table 6. According to the 90° value rule in which materials with contact angles ranging over 90° are thought as hydrophobic, the classification was performed. It was noticed that surface-modified montmorillonite impacts the hydrophobic character of the PU/MMT nanocomposite, not only by the type of modification but also by the quantity of content. The obtained PU matrix water contact angle was 77.55° at 0 min and 75.55° after 2 min, indicating poor hydrophobic characteristics of the material [55]. Each of the used surface-modified montmorillonite types and amount of content resulted in higher contact angle values related to neat PU except the 2% Na^+^ and all TSA nanocomposites. Regardless of the loading, the contact angles for TSA nanocomposites are the lowest and vary from 65.84° to 71.45°. Moreover, the TSA samples have the most significant average contact angle reduction value (ACAR)—6.12% after 2 min—compared to the other types of used MMT. The increase in hydrophilicity could be caused by the high amount of hydrophilic groups presented at the surface of TSA montmorillonite. For this reason, TSA nanocomposites are characterized by increased water absorption [56]. The differences in WCA for samples in each series were probably caused by local nanofiller agglomerates that modify the samples’ properties.

**Figure 7:**
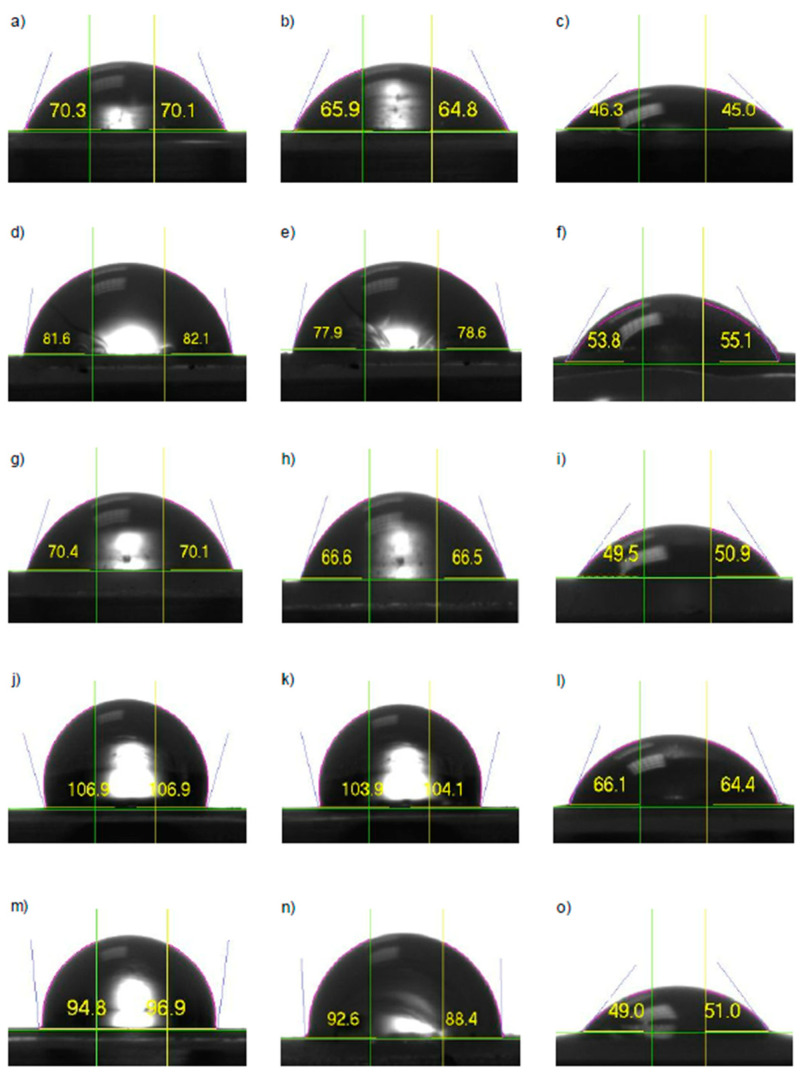
Water and diiodomethane contact angles of selected samples (**a**) WCA0 of neat PU; (**b**) WCA2 of neat PU; (**c**) ICA of neat PU; (**d**) WCA0 of DDA; (**e**) WCA2 of DDA; (**f**) ICA of DDA; (**g**) WCA0 of TSA; (**h**) WCA2 of TSA; (**i**) ICA of TSA; (**j**) WCA0 of OA; (**k**) WCA2 of OA; (**l**) ICA of OA; (**m**) WCA0 of Na^+^; (**n**) WCA2 of Na^+^; (**o**) ICA of Na^+^.

The tendency was observed that with an increase in the nanofiller content, the contact angle increased to a specific value, and after exceeding it, the contact angle value decreased, which is a common phenomenon for nanocomposites [57]. Samples with 1 wt% additions of DDA, OA, and Na^+^ nanofiller provided the best hydrophobicity, which was associated with the best dispersion and possible exfoliation in the polymer matrix (Figure 7). As a result, functional groups present on MMT’s surface may interact and react with hydroxyl groups and isocyanate, which causes improvement in the water resistance of the system. The sample with 1 wt% OA had the highest WCA_0_ (t = 0 min) and WCA_2_ (t = 2 min)_,_ and the lowest ACAR values, indicating the system’s greatest hydrophobicity.

To investigate the interaction between samples with the highest WCA and nonpolarity, each sample with 1 wt% of different nanofillers was tested with diiodomethane (Figure 7). It was noticed that sample swelling occurred for all samples. According to the WCA and ICA analysis, the values of the total energy tension and the dispersive and polar surface energy components were obtained and presented in Table 7. Compared to the neat PU, a sample coded 1% TSA had an almost similar value for the total surface of tension and other components, indicating the insignificant impact of TSA MMT on the intermolecular distribution of forces. The lowest total surface tension was obtained for 1% OA in which the dispersible component was dominant. Thus, the OA MMT addition reduced the number of polar bonds, e.g., hydrogen bonds, between polyurethane chains. It was observed with a decrease of the polar component by almost 99% compared to neat PU. According to the Derlich’s review [58] containing Vogler’s thesis [59], hydrophobic materials are characterized by a water contact angle of *θ* > 65° and a water adhesion tension of *τ* < 30 mN/m. Therefore, by the measured dispersive and polar components, it was confirmed that 1% OA content resulted in hydrophobicity.

### 3.4. X-ray Diffraction (XRD)

**Figure 8:**
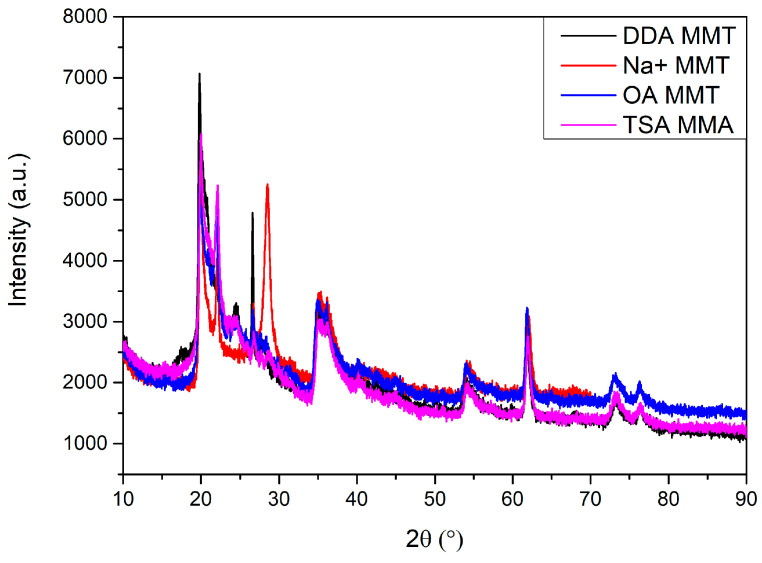
X-ray pattern of surface-modified montmorillonite clays.

**Figure 9:**
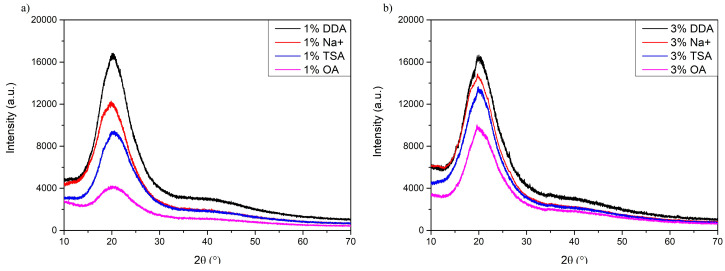
X-ray pattern of surface-synthesized PU/MMT nanocomposites. (**a**) Samples with 1% addition of MMT; (**b**) Samples with 3% addition of MMT.

XRD patterns of surface-modified montmorillonites are presented in Figure 8, and patterns of PU/MMT nanocomposites are presented in Figure 9. All tested montmorillonite clays show almost similar XRD patterns at the tested range. Moreover, the difference between the 2θ of each present peak of patterns does not exceed 0.5°. The most critical peaks for the tested MMTs are present at 2θ of around 19.8°, 22.1°, 35.2°, and 61.8°. The most significant differences are observed at 2θ ≈ 24.5° where the peak of Na^+^-MMT disappeared, at 2θ ≈ 26.6° for DDA-MMT, and at 2θ ≈ 28.5° where the peak appeared only for sodium montmorillonite [60]. The differences noted may be due to a change in the structure of MMT caused by modifications with different compounds.

Analyzing the patterns obtained for the PU/MMT nanocomposites, a broad and intense amorphous peak with a maximum of 2θ = 21° can be observed. This peak may be observed due to the partially ordered structure of the soft and hard segments of the PU matrix [61,62]. Adding different MMTs to the PU matrix changed the intensity and width of the peak. Moreover, it can be observed that each MMT interacts differently with the PU matrix, which is noticeable by the change in intensity and shape of the peak. For the samples from the OA series, the intensity of the peak is the lowest. This may be caused by the alignment of MMT nanoparticles between polymer chains, causing steric hindrances that increase the distance between polymer chains. The stronger the interactions between the matrix and nanofiller are, the greater the intensity of the peaks. This may change microphase separation and the degree of crystallinity of PU/MMT nanocomposites. In addition, the peaks arising from the MMT nanofiller at 2θ > 30° disappeared. This may be caused by structure disruption and the exfoliation of the MMT, which may be evidence of the proper distribution of nanofiller in the PU matrix.

### 3.5. Optical Coherence Tomography (OCT)

Optical coherence tomography (OCT) is an optical method for inner structure evaluation of a broad range of optically scattering materials and devices. It delivers measurement results in the form of 2D cross-sectional tomographic images or 3D volumetric data. The OCT measurements are performed by illuminating the device under test (DUT) with a broadband light source and gathering the backscattered/back-reflected light from inner scattering centers using spatially resolved detection. For this purpose, low-coherence interferometry (LCI), also known as white light interferometry (WLI), was applied. The LCI uses a two-beam optical interferometer like the Michelson or Mach–Zehnder interferometer, combined with a broadband light source. If the detection is performed in the time domain, the interference pattern is recognized for the interfering beams’ optical path difference below the coherence length of the light source. Following that, the backscattering centers inside the DUT are localized with this accuracy. In modern OCT systems, optical detection is performed in the spectral/frequency domain, in which the locations of scattering centers along the optical beam propagation are coded in the frequency of modulation of the recorded optical interference spectrum. Typical OCT systems have a spatial imaging resolution of a few micrometers, with a high measurement speed (thousands of a-scans per second) [63,64,65]. Beyond standard OCT systems, other modalities expand the range of measurements by, e.g., polarization-sensitive (PS-OCT), Doppler-enabled (D-OCT), or spectroscopic (S-OCT) analysis. They all deliver more helpful information about the DUT, like strain field mapping, movement detection inside the sample, or component recognition by the optical features [66,67,68,69].

**Figure 10:**
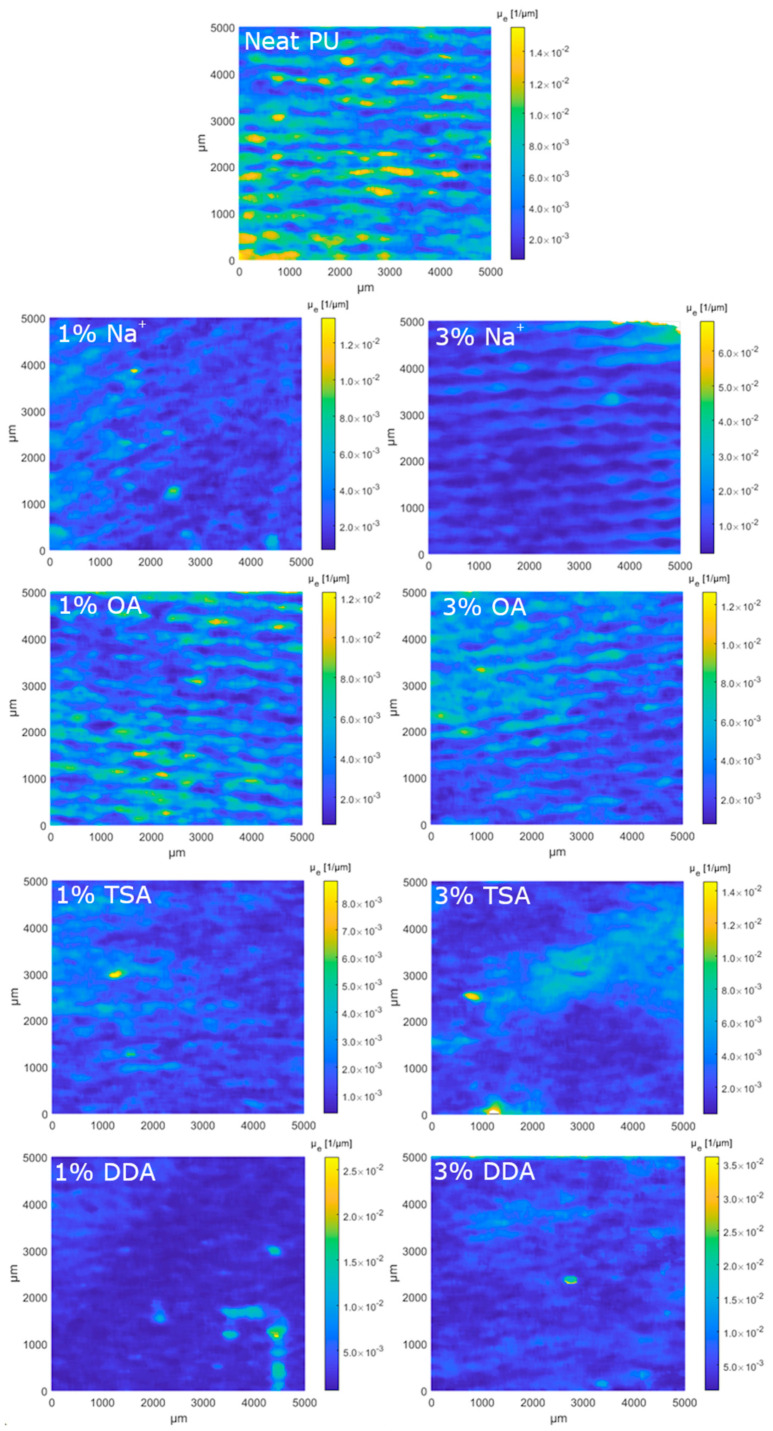
The surface map (according to C-scan plane) of the normalized extinction coefficient.

**Figure 11:**
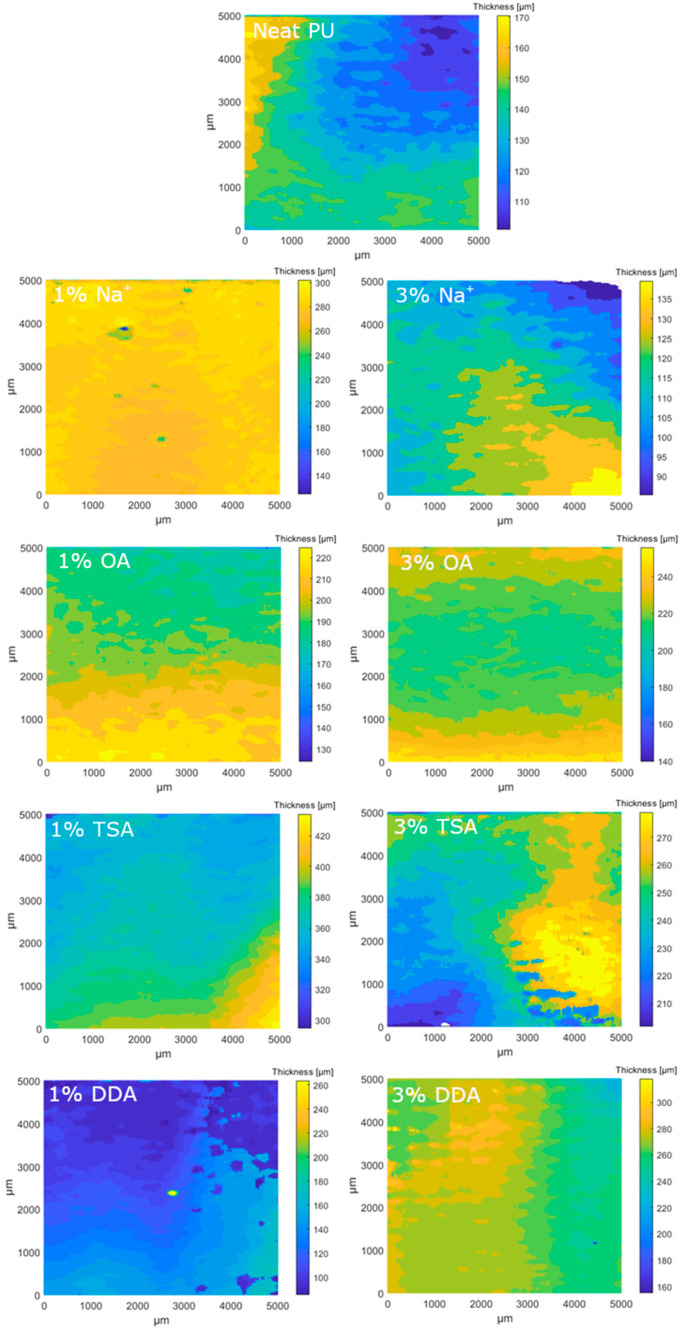
The surface map (according to C-scan plane) of the sample thickness.

**Figure 12:**
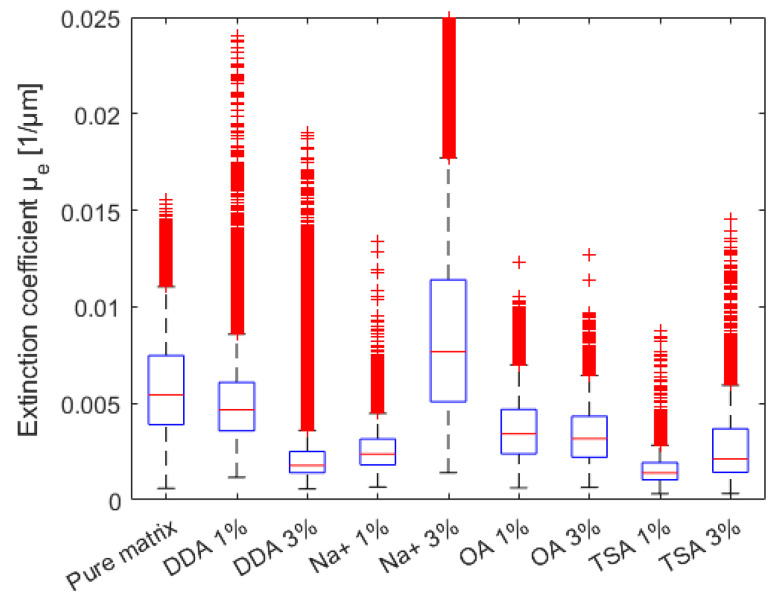
Distribution of the normalized extinction coefficient.

**Figure 13:**
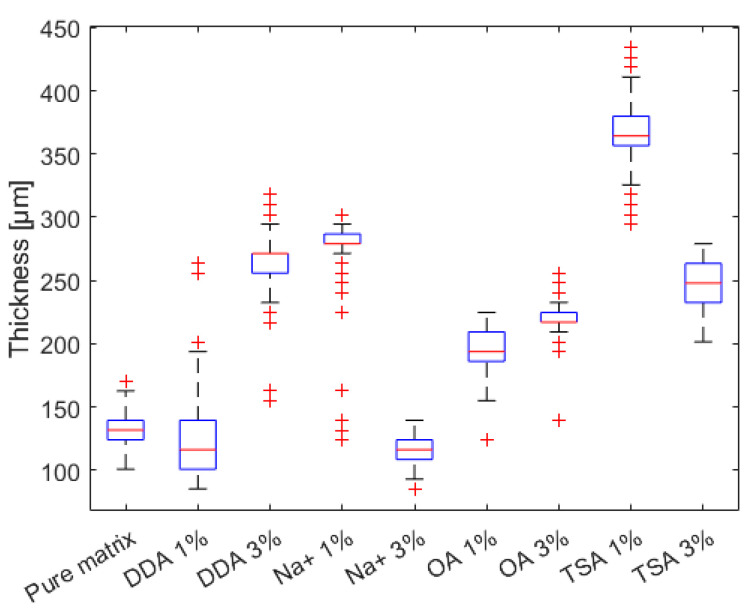
Distribution of the sample thickness.

The OCT measurements were performed in order to estimate the thickness, scattering, and absorption features (expressed as normalized extinction coefficient) of the composites with polyurethane matrixes. The measurements were carried out for the samples with different fillers in two volume concentrations, 1% and 3%, while the pure polyurethan matrix was tested as a reference. OCT is a noncontact and nondestructive measurement technique and does not require any special sample preparation or treatment. The devices under test were cleaned with compressed air and kept in a petri dish during the OCT measurements. All tests were performed at room temperature (23 °C). The local values of the normalized extinction coefficient are presented in Figure 10 and optical thickness in the form of surface maps is illustrated in Figure 11. Their distributions in cumulative form are presented in Figure 11 and Figure 12, respectively. Due to the possible fillers’ influence on the optical scattering and absorption features, expressed as *µ*_e_, the distribution of the filler can be analyzed. For homogeneous materials, the deviation of *µ*_e_ should be very low. Otherwise, if the local filler concentration in the matrix varies, a higher deviation of *µ*_e_ will be noticed. Following the results of µ_e_ analysis (Figure 13), the most homogeneous distribution of the filler is observed for samples coded 3% DDA MMT and 1% TSA MMT.

Comparing the median *µ*_e_ values, the introduction of the filler reduces the *µ*_e_ compared to the pure polymer matrix. The only exception is the 3% Na^+^. Further increasing the dopants’ concentration also increases the scattering and absorption effects. The opposite trend is noticed only for DDA and OA samples.

## 4. Conclusions

In this study, polyurethane nanocomposite thin films were manufactured with the addition of 0–3% wt% of four types of surface-modified montmorillonites. Composites were manufactured using the prepolymer method. The PU/MMT nanocomposites were tested to determine the impact of MMT modification on the properties of the nanocomposites. Materials were tested by thermogravimetric analysis (TGA), dynamic mechanical analysis (DMA), contact angle measurement, X-ray diffraction (XRD), and optical coherence tomography (OCT). Thermogravimetric analysis showed a significant influence of MMT on the thermal decomposition process due to the strong interactions between both phases of the nanocomposite and a possible increase of phase separation due to the exfoliation of the MMT structure. Moreover, dynamic mechanical analysis showed a shift in the glass transition temperature into higher temperatures, which suggests an increase in the structural cross-linking density of the nanocomposites and the potential catalytic effect of selected nanofillers. X-ray diffraction analysis suggested that MMT interacts differently with the PU matrix, which is noticeable by this peak’s change in intensity and shape. This effect may be due to the disruption of the MMT and PU structure and the possible exfoliation of the MMT in polymer matrix. OCT allowed for analysis of the *µ*_e_ parameter and the scattering and absorption effects. The conducted analysis showed that each MMT has a different impact on each above-mentioned property.

In summary, this study has demonstrated the possibility of polyurethane/montmorillonite thin-film nanocomposite manufacturing and has several practical implications for the development of flexible protective coatings and sensors. The results have revealed that the MMT surface structure has significant influence on the structure and properties of composites. Moreover, this article underlines the importance of adequately selecting montmorillonite surface structures for specific applications. This could potentially improve the selection of nanofillers, which should interact appropriately with the polymeric matrix. To develop this knowledge, future studies should investigate the impact of MMT structure modification on other properties of PU nanocomposite thin layers, such as mechanical properties, microstructure, barrier properties, and flammability.

## Figures and Tables

**Table 1:** List of key abbreviations.

Full Name	Abbreviation
Polymer nanocomposite	PNC
Polyurethane	PU
Montmorillonite	MMT
Standard sodium montmorillonite	Na^+^ MMT
Surface-modified montmorillonite (contains octadecylamine and aminopropyltriethoxysilane)	OA MMT
Surface-modified montmorillonite (contains trimethyl stearyl ammonium)	TSA MMT
Surface-modified montmorillonite (contains dimethyl dialkyl (C14–C18) amine)	DDA MMT
Optical coherence tomography	OCT
Dynamic mechanical analysis	DMA
Thermogravimetric analysis	TGA
Water contact angle	WCA
Diiodomethane contact angle	ICA
Average contact angle reduction	ACAR
X-ray diffraction analysis	XRD

**Table 2:** Substrates for manufacturing of PU and PU nanocomposites.

Substrate	Producer	Properties/Additional Information
α,ω-oligo(ethylene-butylene adipate) (Polios 55/20)	Purinova Sp. z o.o., Bydgoszcz, Poland	*M_n_*~2000 g/mol
Methylene diphenyl diisocyanate	Borsodchem, Kazincbarcika, Hungary	Industrial-standard pure 4,4′-MDI stabilized against oxidation, %NCO~33.4 m/m%; functionality~2.0
1,4-butanediol (BDO)	BASF, Ludwigshafen, Germany	Chain extender
1,4-Diazabicyclo [2.2.2] octane	Sigma Aldrich, Warszawa, Poland	Catalyst
Cloisite^®^ Na+ Nanoclay (Na^+^ MMT)	BYK Additives & Instruments, Wesel, Germany	Standard sodium montmorillonite
Montmorillonite Nanoclay, surface-modified (682632) (OA MMT)	Sigma-Aldrich, Saint Louis, MO, USA	Contains 15–35 wt% octadecylamine, 0.5–5.0 wt% aminopropyltriethoxysilane
Montmorillonite Nanoclay, surface-modified (682608) (TSA MMT)	Sigma-Aldrich, Saint Louis, MO, USA	Contains 25–30 wt% trimethyl stearyl ammonium
Montmorillonite Nanoclay, surface-modified (682624) (DDA MMT)	Sigma-Aldrich, Saint Louis, MO, USA	Contains 35–45 wt% dimethyl dialkyl (C14–C18) amine

**Table 3:** The OCT system features [42,43].

Test Parameter	Details
Light source type	20 kHz swept-source laser
Average output power	10 mW
Frame rate	>4 fps
Max. depth imaging range/transverse imaging range	7 mm/10 mm
Central wavelength	1290 nm
Wavelength range	140 nm
Axial resolution (in the air)	12 µm
Lateral resolution	15 µm

**Table 4:** Results of thermogravimetric analysis.

Sample	% Mass Loss Temperature [°C]			*T*_max1_ [°C]	*T*_max2_ [°C]	*T*_max3_ [°C]	Char Residue [%]	Calculated Amount of Rigid Segments [%]
	*T*_2%_	*T*_5%_	*T*_50%_					
Neat PU	271.7	291.2	375.5	303.9	359.9	390.7	7.59	9.2 *
1% Na^+^	285.9	302.7	398.6	-	329.5	408.3	7.79	34.9
2% Na^+^	290.9	305.3	396.7	-	331.9	406.0	9.21	35.2
3% Na^+^	287.4	302.7	398.9	-	331.6	407.5	8.48	34.4
1% DDA	289.3	303.2	390.0	-	332.2	401.7	6.40	35.5
2% DDA	286.6	301.2	388.4	-	329.5	401.8	6.71	34.7
3% DDA	288.2	302.4	387.1	-	329.9	394.5	8.32	33.4
1% TSA	289.1	303.5	396.0	-	331.2	407.2	6.99	35.8
2% TSA	289.6	304.6	397.5	-	332.2	406.9	6.75	35.1
3% TSA	293.5	305.7	396.7	-	328.9	405.8	9.21	34.6
1% OA	284.3	301.4	396.3	-	331.9	408.7	6.76	36.1
2% OA	287.6	303.9	395.2	-	332.5	405.9	7.04	35.4
3% OA	284.3	300.1	387.9	-	324.4	399.5	7.60	34.5

* The result has a large error due to the difficulties in deconvolution of peaks.

**Table 5:** Results of dynamic thermal analysis of composites and their components.

Sample	*T*_g_ [°C]	*E*’ at *T*_1_ = −60 °C [MPa]	*E*’ at *T*_2_ = 0 °C [MPa]	Tan *δ* at *T*_g_ [-]
Neat PU	−28.64	2469	58	0.379
1% Na^+^	−25.52	3439	102	0.355
3% Na^+^	−25.52	2516	52	0.377
1% DDA	−27.27	2476	56	0.376
3% DDA	−22.60	2708	21	0.425
1% TSA	−25.32	3294	63	0.410
3% TSA	−14.43	3432	50	0.445
1% OA	−28.37	2760	49	0.361
3% OA	−27.27	2824	79	0.339

**Table 6:** Wetting properties: water contact angle at 0 min (WCA_0_), water contact angle at 2nd min (WCA_2_), average contact angle reduction after 2 min (ACAR), *T* = 21 °C.

Sample	WCA_0_ [°]	WCA_2_ [°]	ICA [°]	Adhesion Work_0_ [mJ/m^2^]	Surface Energy [mN/m^2^]	ACAR [%]
Neat PU	77.55 ± 1.40	75.55 ± 1.00	49.07 ± 3.18	88.50 ± 1.74	37.02 ± 0.88	2.58
0.5% TSA	66.87 ± 0.68	60.73 ± 2.08	-	101.40 ± 0.79	43.64 ± 0.42	6.12
1% TSA	71.45 ± 1.33	67.93 ± 1.47	52.31 ± 4.49	95.96 ± 1.61	40.81 ± 0.82	
2% TSA	70.28 ± 1.66	66.79 ± 3.19	-	97.35 ± 1.98	41.54 ± 1.03	
3% TSA	65.84 ± 2.91	62.29 ± 3.03	-	102.57 ± 3.38	44.28 ± 1.79	
0.5% DDA	77.14 ± 3.78	74.57 ± 3.27	-	88.98 ± 4.68	37.26 ± 2.36	3.26
1% DDA	83.50 ± 1.47	80.61 ± 2.02	52.35 ± 2.54	81.04 ± 1.85	33.29 ± 0.92	
2% DDA	80.22 ± 2.50	78.40 ± 3.29	-	85.16 ± 3.12	35.34 ± 1.57	
3% DDA	76.29 ± 2.11	73.25 ± 1.80	-	90.04 ± 2.60	37.80 ± 1.31	
0.5% OA	94.80 ± 3.85	89.52 ± 5.37	-	66.71 ± 4.86	26.25 ± 2.37	3.71
1% OA	107.39 ± 1.34	104.73 ± 1.20	64.07 ± 2.44	51.05 ± 1.62	18.62 ± 0.79	
2% OA	97.68 ± 2.25	94.19 ± 2.05	-	63.07 ± 2.83	24.47 ± 1.38	
3% OA	98.16 ± 4.65	95.01 ± 5.72	-	62.49 ± 5.83	24.18 ± 2.84	
0.5% Na^+^	74.80 ± 4.16	71.12 ± 5.35	-	91.85 ± 5.07	38.73 ± 2.59	3.77
1% Na^+^	97.36 ± 3.54	93.07 ± 3.90	48.03 ± 1.63	63.48 ± 4.45	24.67 ± 2.17	
2% Na^+^	68.30 ± 2.15	65.74 ± 1.78	-	99.71 ± 2.54	42.76 ± 1.33	
3% Na^+^	86.89 ± 2.15	85.17 ± 2.05	-	76.74 ± 2.73	31.17 ± 1.34	

**Table 7:** Dispersive and polar components of the surface energy of selected samples.

Sample	Total Surface Tension [mN/m]	Dispersibility [mN/m]	Polarity [mN/m]
Neat PU	41.06 ± 1.69	34.78 ± 1.77	6.28 ± 2.68
1% TSA	41.77 ± 2.30	32.96 ± 2.55	8.80 ± 0.38
1% DDA	36.58 ± 2.67	33.19 ± 1.94	3.39 ± 0.74
1% OA	25.50 ± 0.16	25.44 ± 0.21	0.06 ± 0.05
1% Na^+^	35.76 ± 0.59	35.36 ± 0.90	0.39 ± 0.36

## Data Availability

The data presented in this study are available in the Influence of montmorillonite clay surface modification on the properties of elastomeric thin layer nanocomposites. Additional data can be sent upon request.

## Supplementary Materials

The following supporting information can be downloaded at: https://www.mdpi.com/article/10.3390/ma16041703/s1, Figure S1: Thermogravimetric (TG) curve of Poles 55/20; Figure S2: DTG curve of Poles 55/20.
