# Supplementary material for: Influence of Surface-Modified Montmorillonite Clays on the Properties of Elastomeric Thin Layer Nanocomposites
Source: Materials (Basel). 2023 Feb 17;16(4):1703. doi: 10.3390/ma16041703 (PMC9964914; doi:10.3390/ma16041703)
Supplement: Supplementary file 1 [file materials-16-01703-s001.zip › materials-2166837-supplementary.pdf]

materials

Supplementary data

Influence of Surface Modified Montmorillonite Clays on the Properties of Elastomeric Thin Layer Nanocomposites

Adam Olszewski [1,*], Aleksandra Ławniczak [1], Paulina Kosmela [1], Marcin Strąkowski [2], Aleksandra Mielewczyk-Gryń [3], Aleksander Hejna [4], Łukasz Piszczyk [1]

1 Department of Polymer Technology, Chemical Faculty, G. Narutowicza St. 11/12, Gdansk University of Technology, 80-233 Gdansk, Poland; adam.olszewski@pg.edu.pl (A.O.), lawniczak.contact@gmail.com (A.Ł.), paulina.kosmela@pg.edu.pl (P.K.), lukasz.piszczyk@pg.edu.pl (Ł.P.)
2 Department of Metrology and Optoelectronics, Faculty of Electronics, Telecommunications and Informatics, Gdańsk University of Technology, 11/12 G. Narutowicza Str., 80-233 Gdańsk, Poland; marcin.strakowski@pg.edu.pl (M.S.)
3 Faculty of Applied Physics and Mathematics, Gdańsk University of Technology, Narutowicza 11/12, 80-233 Gdańsk, Poland; alegryn@pg.edu.pl (A.M.G.)
4 Institute of Materials Technology, Poznan University of Technology, Piotrowo 3, 61-138 Poznań, Poland; aleksander.hejna@put.poznan.pl (A.H.)
* Correspondence: adam.olszewski@pg.edu.pl

Citation: Olszewski, A.; Ławniczak, A.; Kosmela, P.; Strąkowski, M.; Mielewczyk-Gryń, A.; Hejna, A.; Piszczyk, Ł. Influence of Surface-Modified Montmorillonite Clays on the Properties of Elastomeric Thin Layer Nanocomposites. *Materials* **2023**, *16*, 1703. https://doi.org/10.3390/ma16041703

Academic Editors: Ivan Chodák and Hamed Peidayesh

Received: 31 December 2022
Revised: 3 February 2023
Accepted: 15 February 2023
Published: 17 February 2023

Copyright: © 2023 by the authors. Licensee MDPI, Basel, Switzerland. This article is an open access article distributed under the terms and conditions of the Creative Commons Attribution (CC BY) license (https://creativecommons.org/licenses/by/4.0/).

1. Thermogravimetric analysis of Poles 55/20

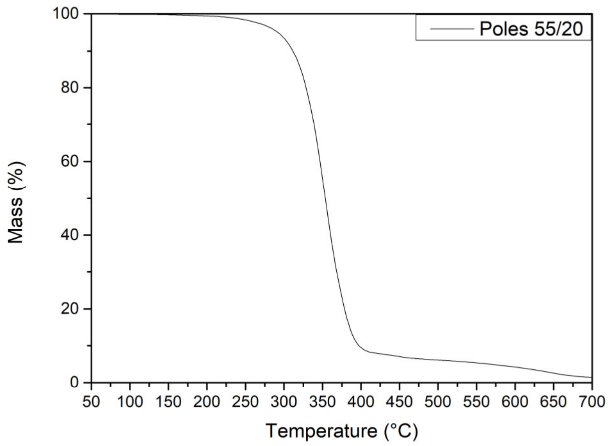

Figure S1. Thermogravimetric (TG) curve of Poles 55/20

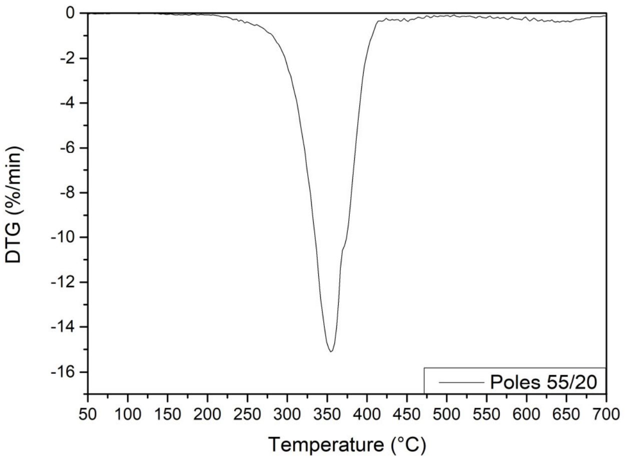

Figure S2. DTG curve of Poles 55/20
